# Hypothermia Reduces Toll-Like Receptor 3-Activated Microglial Interferon-**β** and Nitric Oxide Production

**DOI:** 10.1155/2013/436263

**Published:** 2013-03-25

**Authors:** Tomohiro Matsui, Yukari Motoki, Yusuke Yoshida

**Affiliations:** ^1^Department of Laboratory Sciences, Yamaguchi University Graduate School of Medicine, 1-1-1 Minami-kogushi, Ube, Yamaguchi 755-8505, Japan; ^2^ACEL, Inc., SIC1 1201, 5-4-21 Nishihashimoto, Midori-ku, Sagamihara, Kanagawa 252-0131, Japan

## Abstract

Therapeutic hypothermia protects neurons after injury to the central nervous system (CNS). Microglia express toll-like receptors (TLRs) that play significant roles in the pathogenesis of sterile CNS injury. To elucidate the possible mechanisms involved in the neuroprotective effect of therapeutic hypothermia, we examined the effects of hypothermic culture on TLR3-activated microglial release of interferon (IFN)-**β** and nitric oxide (NO), which are known to be associated with neuronal cell death. When rat or mouse microglia were cultured under conditions of hypothermia (33°C) and normothermia (37°C) with a TLR3 agonist, polyinosinic-polycytidylic acid, the production of IFN-**β** and NO in TLR3-activated microglia at 48 h was decreased by hypothermia compared with that by normothermia. In addition, exposure to recombinant IFN-**β** and sodium nitroprusside, an NO donor, caused death of rat neuronal pheochromocytoma PC12 cells in a concentration-dependent manner after 24 h. Taken together, these results suggest that the attenuation of microglial production of IFN-**β** and NO by therapeutic hypothermia leads to the inhibition of neuronal cell death.

## 1. Introduction

Toll-like receptors (TLRs) are major sensors of pathogen-associated molecular patterns (PAMPs) that mediate innate immunity and are involved in adaptive immune responses [1]. Production and release by damaged cells of molecules that are abnormally expressed or whose structures are altered can stimulate the activity of TLRs [2, 3]. Under these conditions, these molecules are recognized as damage- or danger-associated molecular patterns that trigger immediate responses or enhance reactions to tissue injury and inflammation [3–5].

Microglia express TLRs and are principal immune cells in the central nervous system (CNS). Their functional characteristics have received much attention because these cells represent the major source of immune mediators in the brain [6]. Although stimulation of TLRs in microglia activates functions that are important for the elimination of pathogens [7], microglial TLRs, particularly TLR2 and TLR4, mediate stroke-induced injury to the CNS [8, 9], neuroinflammation, and neuronal damage [4, 5, 10, 11] by responding to endogenous compounds. Lehnardt et al. investigated in detail the role of endogenous mechanisms that trigger activation of microglial TLRs [4]. They found that the molecular chaperon, heat shock protein 60 (HSP60), serves as a signal of CNS injury by activating microglial signal pathways mediated by TLR4 and the TLR adapter protein called myeloid differentiation factor 88 (MyD88). Dying CNS cells release HSP60 that binds to microglia, which in turn secrete neurotoxic nitric oxide (NO). These data provided the first evidence for an endogenous pathway that may be common to many forms of neuronal injury and that bidirectionally links CNS inflammation with neurodegeneration. Lehnardt et al. [4] characterized these events as a “vicious cycle of neurodegeneration,” in which the initial cause of CNS cell death, irrespective of its nature, leads to the release of endogenous molecules from dying cells, which activate microglia *via* TLRs. This leads to the release of neurotoxic molecules that cause further injury to neighboring neurons. Consistent with this hypothesis, necrotic neurons activate an MyD88-dependent pathway in microglia, leading to the release of not only NO, but also proinflammatory cytokines, such as interleukin (IL)-6 and tumor necrosis factor (TNF)-*α* [5], which are associated with neuronal injury [12, 13].

Therapeutic hypothermia can potentially protect neurons after severe brain damage, such as that occurring after traumatic brain injury (TBI) and cardiac arrest [14, 15]. To elucidate the possible mechanisms responsible for this neuroprotective effect, we and others have examined whether decreasing temperature affects *in vitro* microglial release of inflammatory factors through activation of TLR2 and TLR4 and have demonstrated that the production of microglial TNF-*α*, IL-6, and NO is in fact reduced under hypothermic culture conditions [16–20].

In the present study, we focused on examining the effects of hypothermic culture on the production of interferon (IFN)-*β* and NO by microglia, in which TLR3 was activated, to better understand the relationship between therapeutic hypothermia and microglial responses. TLR3 is a major mediator of cellular responses to viral infection, even in the CNS [21] because it responds to double-strand RNA (dsRNA), a common intermediate of viral replication [22]. This antiviral response is characterized by high expression of type I IFNs, predominantly IFN-*α* and IFN-*β*, which is induced by the stimulation of TLR3 [23]. During inflammation, TLR3 also recognizes RNA released from necrotic cells as an endogenous ligand [24–26], leading to the release of type I IFNs [24, 25]. Thus, host-derived nucleic acids are likely to act as endogenous ligands that activate TLR3, particularly in microglia, which may further amplify inflammation in the CNS.

In the present study, we used a synthetic analog of dsRNA, polyinosinic-polycytidylic acid (poly(I:C)), to stimulate TLR3 signaling. This compound activates microglia *in vitro* [27, 28] and *in vivo* [29–31], and the latter leads to neurodegeneration [29]. Therefore, poly(I:C) can be used to study TLR3-driven neuroinflammation mediated by microglia in the CNS. Poly(I:C) induces type I IFNs [32], IFN-*β* more so than IFN-*α*, in microglia [27]. Despite some anti-inflammatory effects in the CNS, for example, IFN-*β* reduces the expression of proinflammatory cytokines [33, 34] and inhibits the infiltration of T cells [35], it directly induces neuronal cell death [36] and as found for other cytokines, excessive levels or inappropriate activity of type I IFNs can cause toxicity and even death (neurodegeneration) [37, 38]. Thus, the function of IFN-*β* in the CNS is somewhat controversial.

To determine their involvement in neuronal protection induced by hypothermia, we investigated whether IFN-*β* and NO directly induced death of a neuronal pheochromocytoma cell line (PC12).

## 2. Materials and Methods

The Animal Care Committee of Yamaguchi University School of Medicine reviewed and approved all protocols used in this study.

### 2.1. Isolation of Microglia

Microglia were isolated from primary cultures of the brains of 1- to 3-day-old Wistar rats or C57BL/6N mice (purchased from Japan SLC, Hamamatsu, Japan) as described in our previous reports, including removal of the meninges [20, 39]. Cell purity was >95% as determined by flow cytometric analysis and immunocytochemistry staining of the microglial markers, Mac-1 (CD11b) and Iba1, respectively. Both markers are reliable markers for microglia in this conventional method for isolation of these cells with similar purities (90%–99.5%) [5, 40, 41].We used anti-Mac-1 (Immunotech, Marseille, France) and anti-Iba1 (Wako Pure Chemical Industries, Osaka, Japan) antibodies, respectively, for this purpose. In addition, we confirmed that the culture did not contain astrocytes using an antibody against glial fibrillary acidic protein by immunocytochemistry staining, in accordance with the result of another study [40].

### 2.2. Microglial Cell Culture

Rat or mouse microglia (4 × 10^4^ cells/well in untreated 96-well plates) were incubated with or without poly(I:C) (100 *μ*g/mL; Imgenex, San Diego, CA, USA) in Dulbecco's Modified Eagle's Medium (DMEM) (Gibco, Grand Island, NY, USA) containing 10% fetal bovine serum (FBS) (Gibco). Cells were incubated under hypothermia (33°C) and normothermia (37°C) for 48 h to measure the production of IFN-*β* and NO. On the basis of our preliminary investigations on the optimal responses to each variable, the dose of poly(I:C) and incubation period were determined. Cell-free supernatants were stored at −80°C.

### 2.3. IFN-*β* Assay

Concentrations of mouse IFN-*β* present in microglial culture supernatants were measured in duplicate using an enzyme-linked immunosorbent assay (ELISA) kit (PBL Interferon Source, Piscataway, NJ, USA), according to the manufacturer's instructions. We performed IFN-*β* assay on mice because ELISA kit for only mouse IFN-*β* was commercially available.

### 2.4. NO Assay

NO production in rat and mouse microglia was detected and quantified as nitrite (NO_2_^−^), a relatively stable metabolite of NO that accumulates in the culture medium. A colorimetric assay using Griess reagent (Sigma-Aldrich, St. Louis, OH, USA) was performed as previously described [20, 39].

### 2.5. PC12 Cell Culture

The rat PC12 cell line was obtained from the RIKEN BioResource Center (RIKEN, Ibaraki, Japan). The undifferentiated cells were grown at 37°C in DMEM (Gibco) supplemented with 10% FBS (Nichirei Bioscience, Tokyo, Japan) and 10% horse serum (HS) (Gibco).

### 2.6. Cytotoxicity Assay

PC12 cells (2 × 10^3^ cells/well) were placed on type I collagen-coated 96-well plates containing culture medium and incubated for 24 h at 37°C. Thereafter, the culture supernatants were substituted for the conditioned medium by DMEM supplemented with 0.1% FBS, 0.1% HS, and 10 ng/mL mouse nerve growth factor 2.5S (Almone Labs, Jerusalem, Israel), including various concentrations of rat recombinant IFN-*β* (Sigma-Aldrich) or sodium nitroprusside dehydrate (an NO donor) (SNP; Wako Pure Chemical Industries), and the cells were cultured at 37°C for 24 h. The viability of the cultures was determined colorimetrically using WST-8 reagent (Nacalai Tesque, Kyoto, Japan) in a modified 3-(4,5-dimethylthiazol-2-yl)-2,5-diphenyltetrazolium bromide (MTT) reduction assay [42]. In brief, culture supernatants were replaced by the conditioned medium including 10% WST-8 reagent and incubated at 37°C for 1 h. The absorbance was measured at 450 nm using a microplate reader. Cell viabilities are presented as values relative to those obtained when cells were treated with vehicle (0.04% sterile distilled water) to control for variation between experiments.

### 2.7. Statistical Analysis

Data are expressed as mean ± standard  error  of  the  mean  (SEM). Differences in values for two groups or among groups were analyzed using the paired *t*-test or one-way analysis of variance followed by the Newman-Keuls multiple comparison method (StatFlex Ver5.0, Artech, Osaka, Japan). The value of *P* < 0.05 was considered to indicate a significant difference. 

## 3. Results

### 3.1. Effect of Hypothermic Culture on the Production of IFN-*β*

**Figure 1:**
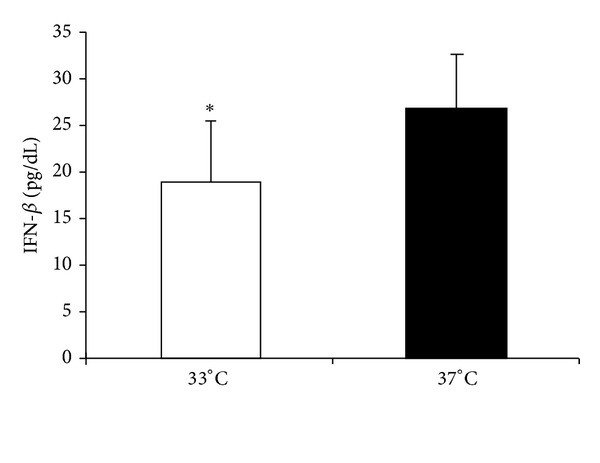
Effect of hypothermic culture on IFN-*β* production by poly(I:C)-stimulated mouse microglia. Mouse microglia (4 × 10^4^ cells/well) were cultured with 100 *μ*g/mL poly(I:C) under hypothermic (33°C) and normothermic (37°C) conditions for 48 h. IFN-*β* levels in culture supernatants were measured by ELISA. Data are expressed as means ± SEM (*n* = 8). *P < 0.05 compared with 37°C.

IFN-*β* was virtually undetectable in unstimulated mouse microglia after 48 h of culture. Application of poly(I:C) to mouse microglia cultured at either 33°C or 37°C induced IFN-*β* production at 48 h (Figure 1). There was a significant reduction in the level of IFN-*β* at 33°C (hypothermia) compared with that at 37°C (normothermia) (Figure 1).

### 3.2. Effects of Hypothermic Culture on the Production of NO

**Figure 2:**
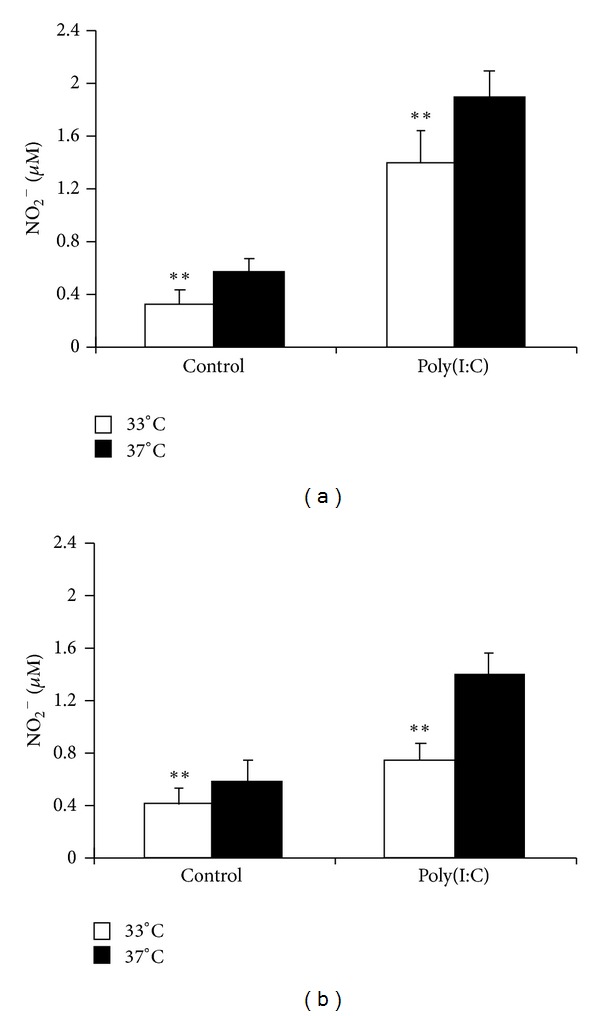
Effects of hypothermic culture on NO production by poly(I:C)-stimulated rat and mouse microglia. Rat (a) and mouse (b) microglia (4 × 10^4^ cells/well) were cultured with or without 100 *μ*g/mL poly(I:C) under hypothermic (33°C) and normothermic (37°C) conditions for 48 h. NO_2_^−^ levels in culture supernatants were measured using a colorimetric assay with Griess reagent. Data are expressed as means ± SEM (*n* = 7 for rat microglia (a) and *n* = 6 for mouse microglia (b)). **P < 0.01 compared with 37°C.

NO_2_^−^ was detected at low levels in an unstimulated rat and mouse microglia after 48 h of culture, and in both cases poly(I:C) increased NO_2_^−^ production (Figures 2(a) and 2(b), resp.). Production of NO_2_^−^ by untreated or treated rat and mouse microglia was reduced by hypothermia compared with normothermia (Figures 2(a) and 2(b), resp.).

### 3.3. Effects of IFN-*β* and SNP, an NO Donor, on the Viability of Neuronal PC12 Cells

**Figure 3:**
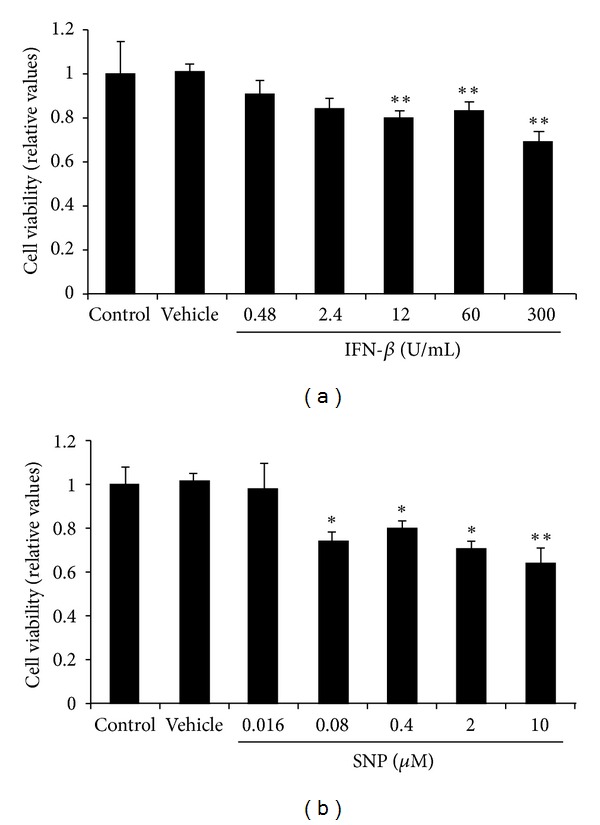
Effects of IFN-*β* and SNP, an NO donor, on the viability of neuronal PC12 cells. Neuronal PC12 cells (2 × 10^3^ cells/well) were treated with or without recombinant IFN-*β* (a) or SNP, an NO donor (b), for 24 h at 37°C. Cell viability was determined using a colorimetric assay with WST-8 reagent as described in the Methods section. Data are presented as values relative to those obtained in cells treated with vehicle and are expressed as means ± SEM (*n* = 5). *P < 0.05, **P < 0.01 compared with vehicle.

IFN-*β* and SNP induced death of neuronal PC12 cells in a concentration-dependent manner 24 h after exposure (Figures 3(a) and 3(b), resp.). These decreases in cell survival were statistically significant at 12–300 U/mL IFN-*β* (80%–69% reduction) and at 0.08–10 *μ*M SNP (79%–63% reduction), compared with vehicle (Figures 3(a) and 3(b), resp.).

## 4. Discussion

TLR signaling can be induced by recognition of either PAMPs or endogenous components. Under pathophysiological conditions, these endogenous agonists are produced or released at unusual concentrations or are present in nonphysiological appearance [43, 44] and trigger immediate responses or enhance reactions to tissue injury and inflammation in microglia [4, 5, 8–11]. Therefore, understanding TLR-driven neuroinflammation in microglia seems to be of particular significance for elucidating the possible mechanisms behind the neuroprotective effects of therapeutic hypothermia. We previously examined the effects of hypothermic culture on the release of inflammatory factors by microglia through activation of TLR2 and TLR4 [19, 20]. Here we focused on the stimulation of TLR3 and showed that, in the TLR3-activated microglia, hypothermia (33°C) reduced the production of IFN-*β* and NO at 48 h of culture. To the best of our knowledge, this is the first paper describing microglial responses to hypothermia using poly(I:C), an activator of TLR3. Reduction of NO levels under hypothermic cultures is consistent with reports regarding activators of TLR2 and TLR4 [16–20].

Increased levels of several proinflammatory cytokines, such as IL-1 and IL-6, and NO are present in cerebrospinal fluid (CSF) after severe head injury in humans [14, 45, 46]. These potentially neurotoxic factors are produced by activated microglia when neurons are destroyed after ischemia or trauma [6, 47], and they are associated with secondary brain damage [13, 48]. Thus, evidence indicates that suppression of the release of these factors by microglia contributes to the neuroprotective effects of therapeutic hypothermia after severe brain damage [14, 15, 49, 50]. In fact, therapeutic hypothermia attenuates the increase in levels of proinflammatory cytokines and NO in the CNS after brain injury [14, 49, 51], and this is associated with a favorable outcome compared with normothermia [14, 49]. Further, hypothermia during severe perinatal asphyxia prevents increases in 3′,5′-cyclic monophosphate (as a marker of NO) in the rat brain. In this study, 100% of the hypothermic rats survived, whereas 70% mortality was observed in the normothermic group [52]. We are not aware of any reports showing that levels of IFN-*β* increase in the CSF after brain injury *in vivo*; however, one animal study indicates production of increased levels of IFN-*α* and IFN-*β* after sterile CNS injury [53]. Despite certain anti-inflammatory effects in the CNS [33–35], IFN-*β* may still be deleterious. The mechanisms responsible for neurotoxic effects of type I IFNs remain unclear; however, some investigators have postulated that the indirect effects of cytokines are mediated by their actions on either peripheral organs or glial cells, for example, type I IFNs induce proinflammatory mediators release from microglial cells [54, 55]. Another possibility is that type I IFNs may exert toxic effects directly on neuronal cells. Gene chip analysis of RNA from a culture of brain cells treated with IFN-*α* indicates that neurons are very responsive target cells for IFNs [56]. Consistent with these findings, IFN-*β* induces death of neuronal cells [36]. Here we found that hypothermia reduced the production of IFN-*β* by microglia expressing activated TLR3. Taken together, our findings suggest that the neuroprotective effects of therapeutic hypothermia are related to the attenuation of the production of NO and IFN-*β* by microglia, although the clinical significance of these findings remains to be determined.

To determine a possible pathophysiological involvement of the decreased production of IFN-*β* and NO by microglia for hypothermic neuronal protection, we examined whether IFN-*β* and/or NO directly induced neuronal PC12 cell death. We were able to demonstrate that IFN-*β* and NO independently decrease cell survival in a concentration-dependent manner, in agreement with a previous study on the effects of NO [57]. To the best of our knowledge, the present study is the first to demonstrate that IFN-*β* induces this effect, although we are aware that it induces apoptosis in a human neuroblastoma cell line [36]. The concentration-dependent IFN-*β*- and NO-induced neuronal cell death and *in vivo* findings of their elevated levels in the CNS after CNS injury [46, 53] support the conclusion that a decrease in their levels during hypothermia contributes toward protection of neurons. The conditioned media from TLR3-activated microglia may have yielded similar direct effects in such experiments; however, we were unsuccessful in our preliminary attempts to answer this question.

Because we used poly(I:C), a synthetic analog of dsRNA, to stimulate TLR3 in microglia, it is possible that activation of this receptor in our present study differs from that in sterile CNS injury. However, poly(I:C) has been used to induce TLR3 signaling to examine its contribution to the pathophysiology of certain noninfectious conditions [29–31]. Nonetheless, it would be of interest to utilize RNAs that act as endogenous TLR3 ligands [24–26] from injured CNS cells [4] in such experiments. It would also be interesting to study other CNS-derived endogenous ligand(s) that colocalize with TLR3 in microglia, such as stathmin, a regulator of microtubules [58], which is present in myelin sheaths and upregulated during neuroinflammation [59]. Further research using these models to confirm our present findings may yield much clinically relevant data. Our study shows that the production of IFN-*β* and NO by microglia was reduced when TLR3 signaling was activated by poly(I:C) under hypothermic culture conditions. Inactivating TLR3 using methods such as RNA interference and/or the use of TLR3-knockout mice would further support the role of TLR3 signaling.

## 5. Conclusions 

Here, we demonstrated that hypothermia reduced the production of IFN-*β* and NO by microglia expressing activated TLR3 and that these factors induced neuronal cell death. Our results suggest that the attenuation of the production of IFN-*β* and NO by microglia induced by therapeutic hypothermia leads to the inhibition of neuronal cell death. Studies of stroke using animal models suggest that activation of TLRs by the release of endogenous ligands contributes to tissue injury and indicates that TLR2 and TLR4 [8, 9, 60, 61], but not TLR3, contribute to this pathological process [62]. Although studies of mice lacking TLR3 support these findings, the activation of TLR3 in microglia by poly(I:C) leads to neurodegeneration [29]. This indicates that expression of TLR3 by microglia plays a role in CNS injury under certain conditions. Our studies here focused on TLR3 signaling in microglia and are the first to show the effects of hypothermia on TLR3-driven neuroinflammation. They reveal a possible mechanism responsible for the neuroprotective effects of therapeutic hypothermia.
