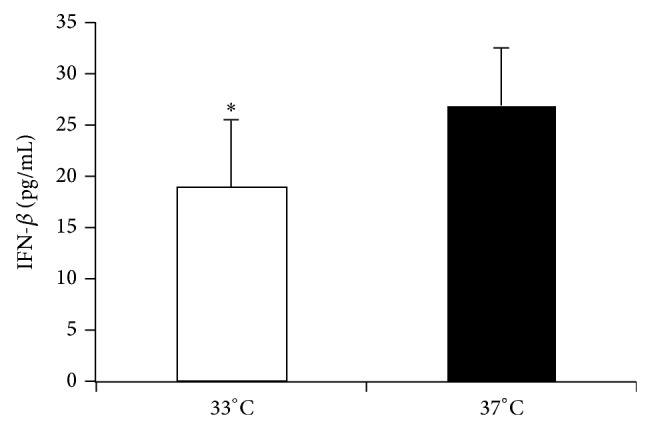# Corrigendum to “Hypothermia Reduces Toll-Like Receptor 3-Activated Microglial Interferon-*β* and Nitric Oxide Production”

**DOI:** 10.1155/2015/136296

**Published:** 2015-03-16

**Authors:** Tomohiro Matsui, Yukari Motoki, Yusuke Yoshida

**Affiliations:** ^1^Department of Laboratory Sciences, Yamaguchi University Graduate School of Medicine, 1-1-1 Minami-kogushi, Ube, Yamaguchi 755-8505, Japan; ^2^ACEL, Inc., SIC1 1201, 5-4-21 Nishihashimoto, Midori-ku, Sagamihara, Kanagawa 252-0131, Japan

In “Hypothermia Reduces Toll-Like Receptor 3-Activated Microglial Interferon-*β* and Nitric Oxide Production,” there was an error in Figure 1. The unit for concentrations of IFN-*β* was pg/dL. Here, we provide the right form of Figure 1.

## Figures and Tables

**Figure 1:**